# Glyphosate-microbial interactions: metagenomic insights and future directions

**DOI:** 10.3389/fmicb.2025.1570235

**Published:** 2025-05-23

**Authors:** Ayabonga Sibalekile, Tesfay Araya, Julio Castillo Hernandez, Elmarie Kotzé

**Affiliations:** ^1^Department of Soil, Crop and Climate Sciences, University of the Free State, Bloemfontein, South Africa; ^2^Department of Integrated Science, University of Huelva, Huelva, Spain

**Keywords:** glyphosate, GM crops, next-generation sequencing, rhizosphere, soil microbial communities

## Abstract

Glyphosate [N-(phosphonomethyl) glycine] is the most widely used systematic non-selective herbicide worldwide. However, there is increasing concern about its potential impacts on soil microbial communities, which play crucial roles in maintaining soil functions, plant health, and crop productivity. While glyphosate can be inactivated in soil through strong sorption, desorption remains a significant challenge as glyphosate residues and metabolites can exert toxicity effects on agroecosystems, particularly by altering microbial diversity and functionality. This review synthesizes current knowledge on glyphosate’s behavior in soils and advancements in metagenomics approaches (including their limitations) to better understand the complex interactions between glyphosate and microbial communities in genetically modified (GM) cropping systems. Glyphosate has demonstrated antimicrobial properties, inhibiting the growth of various bacteria and fungi. Conversely, other studies suggest that glyphosate may enhance microbial richness, promoting the proliferation of potential glyphosate degraders (e.g., *Bacillus*, *Stenetrophomonas*, *Pseudomonas, Sphingomonas,* and *Phenylobacterium*) and N_2_ fixing bacteria (e.g., *Bradyrhizobium*, *Rhizobium*, and *Devosia*) in the bulk soil and rhizosphere of GM crops. These contrasting findings are influenced by factors such as soil types, glyphosate rates, and crop varieties. Moreover, the review highlights that methodological discrepancies, including variations in next-generation sequencing (NGS) platforms and reference databases, contribute significantly to inconsistencies in the literature. These differences stem from varying levels of accuracy or annotation standards in the databases and NGS technologies used. To address these challenges, this study underscores the need for standardized molecular and bioinformatics approaches. Integrating advanced long-read sequencing technologies, such as Oxford Nanopore and PacBio, with compatible reference databases could provide more accurate and consistent analyses of microbial community composition at finer taxonomic levels. Such advancements could improve our understanding of how glyphosate influences the balance between pathogenic microorganisms and plant-growth-promoting microbes in GM cropping systems, ultimately informing sustainable agricultural practices.

## 1 Introduction

Weed control is one of the most vital practices requiring the effective application of herbicides to achieve significant growth and meet target yields in crop production. Glyphosate is the most widely used systematic non-selective herbicide in the world due to its effective weed control in agriculture (Helander et al., 2012). Following the introduction of glyphosate-resistant GM crops such as canola, cotton, maize, and soybeans, the use of glyphosate has surged, and areas planted with conventional crops decreased (James, 2004). The use of glyphosate alongside GM crops has improved weed management and encouraged the adoption of conservation agriculture practices, such as crop diversification and minimal soil disturbance. In most countries, including South Africa, glyphosate has remained a dominant herbicide, particularly for weed control in GM cropping systems. Its global market size has grown significantly in recent years, reaching a value of 7.8 billion US dollars in 2020, with projections indicating substantial growth through to 2027 (Statista, 2024). High amounts of glyphosate applied in agriculture have also brought several environmental and health concerns (FAO, 2017), with the overspray of glyphosate resulting in its widespread presence in aquatic and terrestrial environments (Hanke et al., 2010). Both susceptible and glyphosate-resistant GM crops may suffer injuries from glyphosate exposure, which may weaken physiological disease defences, reduce nutrient uptake and utilization of metals by crops, and interfere with rhizosphere microbial ecology (Gomes et al., 2014; Martinez et al., 2018; Chao et al., 2024).

Glyphosate has several desirable properties that have contributed to its widespread use around the world and has been considered safer than other herbicides (Duke and Powles, 2008). It is the leading herbicide to provide good margins of crop safety in most countries. It is reported that glyphosate has high weed-killing efficiency, lower cost than many other herbicides, low toxicity to mammals, and limited risk of leaching into groundwaters due to high adsorption in soils that has relatively higher clay and soil organic carbon content (Baylis, 2000; Busse et al., 2001; Gimsing et al., 2004; Vereecken, 2005). Under certain conditions, glyphosate can be desorbed and reintroduced into the soil solution, where it may affect microbial communities by altering species composition and functionality (Borggaard and Gimsing, 2008; Kremer et al., 2005). However, in several studies, its use in weed control has been linked to toxicity issues and may cause adverse non-target impacts in agroecosystems (Landry et al., 2005; Relyea, 2005; Siimes et al., 2006; Kanissery et al., 2019). Weed species that are more difficult to control with glyphosate have become more common and have led to the evolution of glyphosate-resistance (GR) weeds (Kremer and Means, 2009). The chemical glyphosate and its metabolites can be found in soil, air, water, as well as groundwater, and food products (Agostini et al., 2020). The presence of glyphosate in food products has generated concerns about glyphosate’s potential toxicological and carcinogenic effects on humans (Niemann et al., 2015; Agostini et al., 2020). There is also growing evidence that glyphosate residues in the soil can have significant impacts on soil microorganisms in agricultural domains (Kremer et al., 2005; Zobiole et al., 2011; Guijarro et al., 2018).

Some soil microbes are susceptible to glyphosate due to their dependence on 5-enolpyruvylshikimate-3-phosphate synthase (EPSPS) in the production of essential aromatic amino acids, while others may have developed resistance mechanisms or alternative metabolic pathways that allow them to endure glyphosate exposure. Due to the critical role played by microorganisms in soil quality through their involvement in biogeochemical and nutrient cycling, long-term soil sustainability, and beneficial effects on plant health, it is crucial to examine the effects of glyphosate on soil microbial communities (Gupta et al., 2000; Prashar et al., 2014). Johnsen et al. (2001) and Bünemann et al. (2006) reported that glyphosate had no or just temporary effects on soil microbial properties utilizing extensive or integrative techniques, such as microbial biomass, enzyme activity, and respiration. Insignificant effects of glyphosate on soil microbial properties could result from functional redundancy where multiple species share similar functions in an ecosystem (e.g., nitrogen (N) fixers). As a result, while short-term glyphosate use may not affect soil functions like N fixation, it can still alter microbial community composition and key functions mediated by specific microbial species (Imfeld and Vuilleumier, 2012). Crucial soil microbial species in agricultural systems that have great potential to improve plant health and have significant effects on crop production have been reported to be at risk from glyphosate treatments. This includes species of phosphate-solubilizing fungi (Salmeron-Santiago et al., 2021), N-fixing bacteria (Sugiyama, 2019), and *Pseudomonas* species (Niculaes et al., 2018).

**Figure 1:**
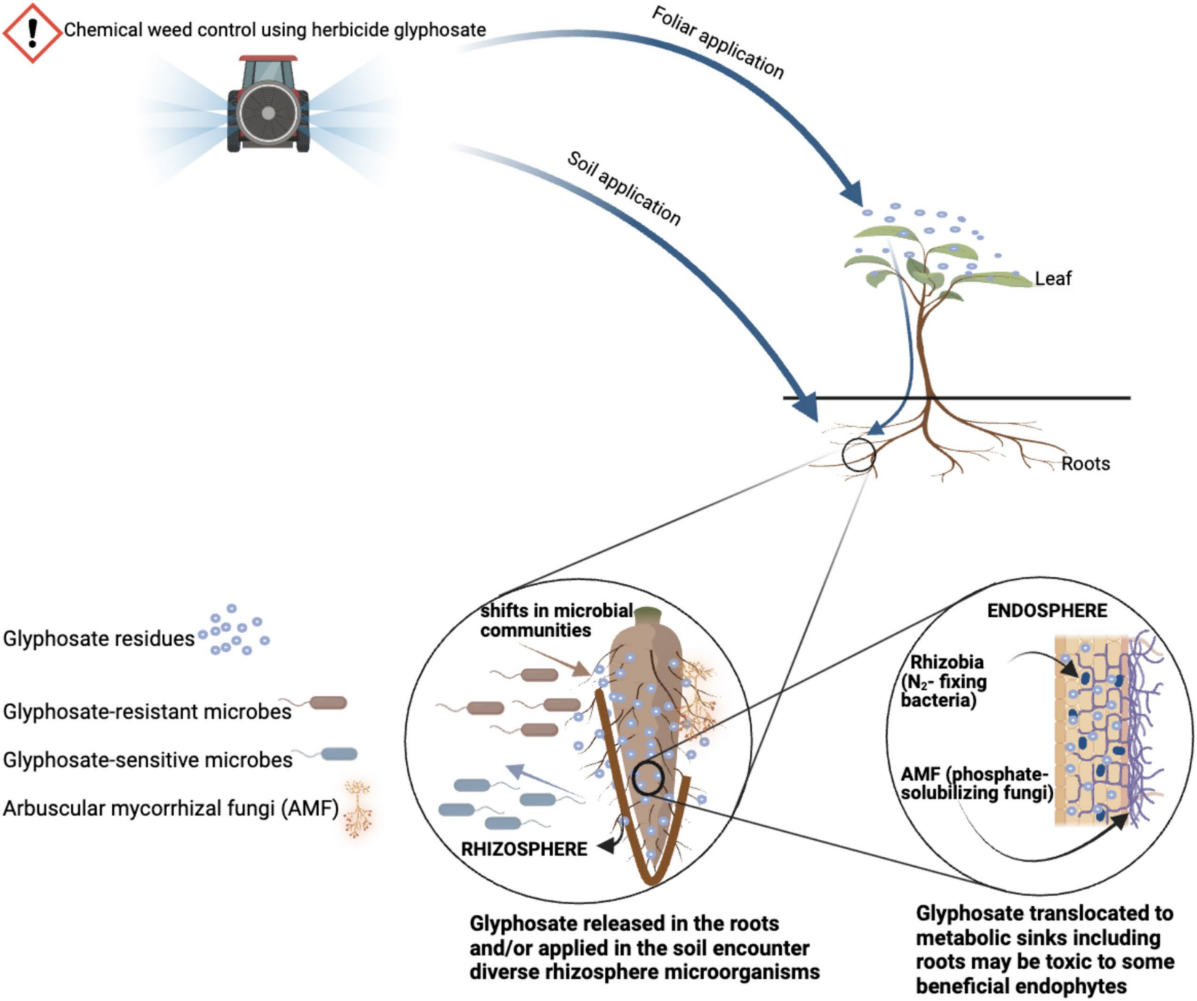
Possible interference of glyphosate with microbial communities in the rhizosphere and endosphere of GM crops. Created with BioRender.com.

Several studies have been investigating glyphosate interactions with soils, plants, and soil microbes to repair glyphosate-contaminated environments and boosting the production of GM crops under glyphosate treatments (Kremer et al., 2005; Meriles et al., 2006; Druille et al., 2016). However, one of the major difficulties in studying glyphosate-plant–soil-microbial interactions is that methods based on culturing and direct morphology observation are time-consuming, often unreliable, and many soil microbes cannot be cultivated in the laboratory. The exact growth requirements of many bacterial and fungal species are unknown, resulting in a sizable portion of the microbial community being unexplored under glyphosate treatments (Qaisrani et al., 2019). To fill this gap in agricultural domains, metagenomics is a current available technique that can directly reveal bacterial, archaeal, and fungal community structures of the bulk soil, rhizosphere, and endosphere microbiota. Metagenomics is the study of all genomic materials (genomes) directly isolated from the environments (Billington et al., 2022). Recent advancements in this field enable the detection of the intricate interactions between soil pathogens and beneficial microbes with various glyphosate treatments in agriculture (Hakim et al., 2018; Almeida and De Martinis, 2019; Pereira et al., 2020; Joshi et al., 2023; Figure 1).

Recent advancements in sequencing technologies, such as Oxford Nanopore R10.4.1, have significantly improved the accuracy of taxonomic profiling at the species level. These improvements are particularly relevant for glyphosate-microbial interaction studies, as they allow for more precise detection of microbial communities in glyphosate-impacted soils (Zhang et al., 2023; Heikema et al., 2020). To conduct this review, we utilized Scopus to search for articles published between 2010 and 2025 using the keywords ‘glyphosate’, ‘soil microbial communities’, and ‘rhizosphere’. The search yielded 343 articles, of which 79 were identified as relevant to the scope of the review. Six of these articles used recent metagenomic approaches to study glyphosate effects on microbial communities in bulk soil. Another 9 articles used recent metagenomic approaches to study glyphosate-microbial interaction on the rhizosphere of GM crops, of which 2 are relevant review articles, and no articles were found that described glyphosate effects on microbial communities and richness in the endosphere of GM crops using recent metagenomic approaches. Most of these studies focused on bacterial communities rather than archaeal and fungal communities, with Illumina technology and SILVA database as the predominant methodologies. Additionally, these studies have yielded conflicting results, where some studies reported no significant effects and others reported an increase in microbial richness and shifts in microbial communities following glyphosate treatments in bulk soil and rhizosphere of glyphosate-resistant GM crops.

Therefore, this review aims to point out the current knowledge of glyphosate’s behavior in soils and the advancements in metagenomics (including their limitations) contributing to the complete comprehension of the intricate interactions between glyphosate, plants, soils, and microbes within the rhizosphere and endosphere of GM crops. The understanding of glyphosate-plant–soil-microbial interactions is the key progression in designing diagnostic methods for glyphosate toxicity and boosting plant production of GM crops.

## 2 Glyphosate’s mode of action during weed control

Glyphosate, which is the active ingredient in Roundup and other weedkilling formulations, is a non-selective herbicide; it can kill all plant species, although there is variation between plant species regarding levels of natural tolerance (Duke et al., 2012). Its mode of action is to inhibit EPSPS, a key enzyme in the synthesis of aromatic amino acids (phenylalanine, tryptophan, tyrosine) in plants and some fungi, and bacteria (Tan et al., 2006). Without these amino acids, plants cannot make the required proteins which are precursors of numerous natural products (e.g., pigments, alkaloids, hormones, and cell wall components), resulting in the death of susceptible plants. Glyphosate-resistant GM crops consist of a resistant EPSP gene of bacterial (*Agrobacterium tumefaciens*) origin which is commonly known as CP4-EPSPS, and it confers a high level of resistance in GM crops (Patterson et al., 2018). Also, in GM plants the EPSPS enzyme is overproduced, allowing glyphosate-resistant plants to successfully produce aromatic amino acids when glyphosate is applied (Funke et al., 2006).

Glyphosate does not rapidly disrupt the shikimate pathway. The herbicide translocates throughout the plant system and kills it effectively by limiting the synthesis of aromatic amino acids and exposing the plant to disease. For example, the earliest symptoms of herbicide damage may take several days to appear, with plants usually dying within 10 to 20 days (Tan et al., 2006). With the use of GM cultivars, glyphosate can be applied after emergence, through flowering, controlling a variety of annual and perennial weeds without endangering GM crops (Coulter and Nafziger, 2007). There are a variety of criteria that farmers must consider when determining the ideal time to remove weeds with glyphosate in GM crops. Weeds start to compete with crops during early growth stages, especially when the soil has insufficient water, nutrients, and light to fully support both crops and weeds in the same field (Glauninger and Holzner, 1982). As a result, glyphosate is more efficient when used on weeds in their early phases of growth (Krausz et al., 2001). Weeds that appear after the first glyphosate spray can be controlled by a second application (Nelson and Renner, 1999).

## 3 Glyphosate interactions with soil

There are various processes and reactions that take place between herbicides and soils that affect the persistence, activity, behavior, and degradation of herbicides. When glyphosate enters the soil, whether through direct application, root exudation from glyphosate-resistant or susceptible crops, glyphosate-treated residues, or decomposing plant tissue, it undergoes various processes including sorption, desorption, mobility, and degradation (Duke et al., 2012).

### 3.1 Sorption and desorption of glyphosate

Glyphosate is easily sorbed onto soil minerals with variable charges, such as iron oxide, aluminium oxides, aluminium silicates, and goethite. To a lesser extent, it also binds to the Fe-oxide coatings of permanent charge minerals (illite, smectite, and vermiculite) and to organic carbon (Duke et al., 2012). Soil rich in variable charge minerals sorb glyphosate effectively and thus reduce its mobility, whereas those dominated by permanent charge minerals exhibit lower sorption capacity (Prata et al., 2003). Although strong sorption can initially inactivate glyphosate, it may later become bioavailable again in the soil through desorption (Gómez Ortiz et al., 2017; Padilla and Selim, 2019). Desorption of glyphosate depends on soil type and it ranges from 5–24% of the initially sorbed glyphosate (Duke et al., 2012; Gómez Ortiz et al., 2017), and can be enhanced by the addition of phosphate (PO_4_^3−^), which competes for sorption sites (Prata et al., 2003; Borggaard and Gimsing, 2008). Overall, sorption–desorption dynamics of glyphosate are influenced by soil properties such as clay content, cation exchangeable capacity (CEC), and soil pH (Cruz et al., 2007; Dollinger et al., 2015; Okada et al., 2016; Gómez Ortiz et al., 2017). Sorption tends to be stronger in acidic soils with lower pH and weaker in alkaline soils, where higher negative charge (or decreased positive sorbent charge) reduces the retention of phosphonates and phosphates (Borggaard and Gimsing, 2008). While strong sorption and low desorption generally limit glyphosate’s availability for microbial degradation, water contamination, metal interactions, and disruption of beneficial soil microbes (Borggaard and Gimsing, 2008; Duke et al., 2012), it is important to recognize that soil degradation capacities vary significantly (Mamy et al., 2005; Sørensen et al., 2006), and desorption remains a major concern due to the potential toxicity of its residues and its metabolites to agroecosystems.

Moreover, glyphosate can also influence various soil properties, particularly chemical characteristics, which in turn may indirectly modulate the composition of soil microbial communities (Romano-Armada et al., 2017). This occurs through its chelating effect, whereby glyphosate binds essential macro-and micronutrients that are critical for soil microbial activity and plant development. C and N minerilisation, along with P cycling are some of the chemical parameters reported to be affect by glyphosate applications (Haney et al., 2002; Chávez-Ortiz et al., 2022). For example, a study by Obour et al. (2016) observed that after 15 years of glyphosate application, soil nitrate (NO_3_^−^) was reduced while levels of available soil P, potassium (K), and Fe, sulfate (SO_4_^2−^) increased. No significant glyphosate effects on soil pH, SOM, exchangeable Ca, Mg, Mn or zinc (Zn) were reported in that study. In contrast, De Mesquita et al. (2023) observed that application of glyphosate (Roundup Promax) increased soil NO_3_^−^ content and decreased soil pH and Ca content. Impacts of glyphosate on soil properties may be context-dependent, shaped by local environmental conditions and soil management practices. Therefore, further research across diverse locations and management systems, incorporating a broader range of soil parameters, is needed to better understand how glyphosate modulates microbial community structures through its effects on soil chemical characteristics.

### 3.2 Microbial degradation of glyphosate in the soil

**Figure 2:**
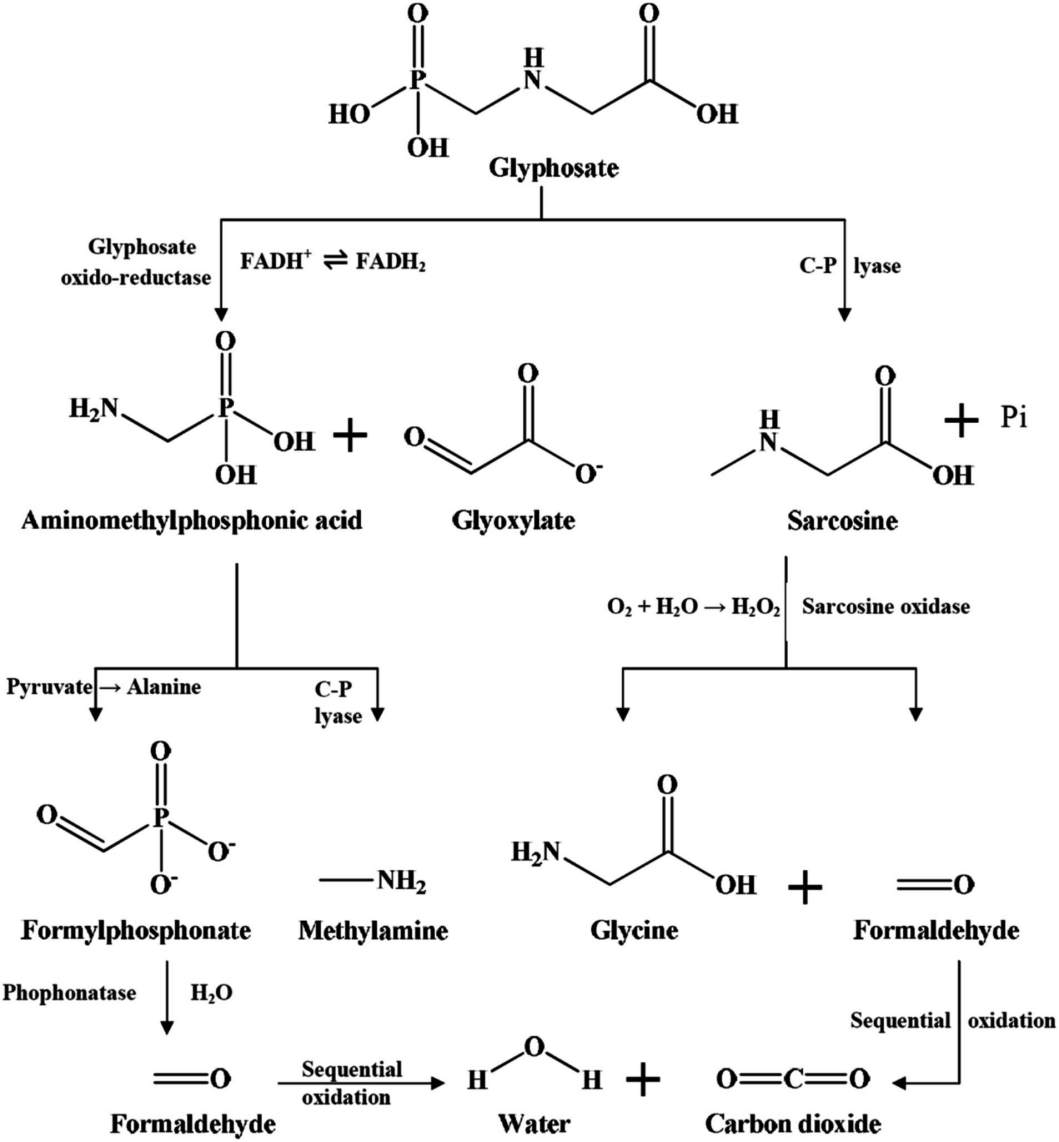
Biodegradation pathways of glyphosate in the soil (Singh et al., 2020b).

Glyphosate can be broken down through absorption, photolysis, thermolysis, as well as soil microbial degradation by catabolic enzymes. Microbial degradation by catabolic enzymes is considered the major process of glyphosate breakdown in soils (Yang et al., 2015). Soil microorganisms have metabolic pathways capable of breaking down glyphosate (Singh et al., 2020a). Two primary glyphosate degradation pathways have been identified in soil (Duke, 2011; Sviridov et al., 2015; Figure 2).

The first pathway is the formation of the sarcosine and inorganic phosphate via the C-P lyase by cleaving the C-P bond (Figure 2). The C-P bond of glyphosate can also be broken down non-enzymatically in the presence of manganese oxide (Barrett and McBride, 2005; Li and Jaisi, 2019). However, the degradation of glyphosate is primarily carried out by soil microorganisms. The second pathway is the formation of aminomethylphosphonic acid (AMPA) and glyoxylate by splitting the glyphosate C-N bond by the enzyme glyphosate oxidoreductase (GOX) to aminomethylphosphonic acid (AMPA) and glyoxylate (Figure 2). The degradation of AMPA is also important because, when released into the environment, AMPA can be toxic and contribute to secondary pollution (Singh et al., 2020a). AMPA can be metabolized to methylamine and phosphate by the C-P lyase found in some soil microorganisms, e.g., *Arthrobacter atrocyaneus* ATCC 13752 (Pipke and Amrhein, 1988), *Ochrobactrum anthropic* GDOS (Hadi et al., 2013), *Stenotrophomonas pavanii* MY01 (Zhao et al., 2024), etc. It can also be metabolized to phosphoryl formaldehyde by transaminase found in *Aspergillus ochraceus* GPK3 and then converted to phosphate and formaldehyde by phosphatase (Sviridov et al., 2012). However, little information is available about soil microorganisms capable of degrading AMPA. In laboratory studies, there are several bacteria and fungi identified to successfully degrade glyphosate in culture media. The best-characterized bacteria belong to the genera *Achromobacter*, *Agrobacterium*, *Bacillus*, *Enterobacter*, *Ochrobactrum*, and *Pseudomonas* (Sviridov et al., 2015; Singh et al., 2020b; Rossi et al., 2021). Among fungi, glyphosate-degrading species primarily found within the genera *Aspergillus*, *Fusarium*, *Penicillium*, and *Trichoderma* among others (Masotti et al., 2023; Singh et al., 2020b).

There are several reports about glyphosate being utilized as a source of phosphorus (P) by many bacteria and fungi (Ternan et al., 1998; Matys et al., 2001; Sviridov et al., 2015). For soil microorganisms to utilize glyphosate or AMPA as a source of P, a C-P lyase is necessary. The first bacteria discovered to be capable of utilizing glyphosate as a source of P was *Pseudomonas* sp. strain PG2982 (Fitzgibbon and Braymer, 1988; Moore et al., 1983). Then followed the discovery of several bacteria isolated from glyphosate-contaminated soil such as *Rhizobiaceae meliloti* strain 1021 (Liu et al., 1991), *Bacillus cereus* strain CB4 (Fan et al., 2012), *Achromobacter* sp. strains MPK 7A (Ermakova et al., 2017), *Comamonas odontotermitis* strain P2 (Firdous et al., 2020). Fewer fungi from the soil have been discovered to utilize glyphosate as a P source; for example, *Aspergillus niger*, *Mucor* IIIR, *Penicillium* IIR, *Scopulariopsis* sp., and *Trichoderma harzianum* (Krzyśko-Łupicka and Orlik, 1997).

Microbial degradation of glyphosate can be rapid during intracellular inorganic P (Pi) shortages, particularly under P deficiency conditions, although such situations are uncommon in natural environments (Sviridov et al., 2015). For example, Pi concentrations have been reported to slow glyphosate biodegradation on several isolates such as *Pseudomonas* sp. PG2982 and *Pseudomonas* sp. GLC11 (Zhan et al., 2018). The ability of isolates to degrade glyphosate effectively, considerably decreases in natural environments due to the rapid response of soil microorganisms to environmental changes, including variations in soil pH, physical and chemical composition, temperature, and nutritional status (Chen et al., 2022). To address this issue, it is important to understand both soil conditions and the origin and ecology of soil microorganisms responsible for glyphosate persistence, mobility, and degradation. This knowledge may support the isolation and development of microbial strains capable of effectively biodegrading glyphosate residues under natural environmental conditions.

## 4 Glyphosate impacts on plant-growth-promoting-microbes on GM crops

Plants naturally host distinct microbial communities outside and inside their tissues in the rhizosphere and endosphere. Rhizosphere is the soil surrounding plant roots and is considered a hotspot for microbial colonization and activity, as it offers a natural microhabitat for a diverse range of microorganisms (Philippot et al., 2013). Endosphere is the interior of plant tissues where microorganisms, including bacteria and fungi, live within the plant’s cells. These microorganisms are known as endophytes, as they colonize the plant’s internal tissues and form a distinct community within the root system. Microorganisms associated with the rhizosphere and endosphere perform a variety of advantageous functions for their host, including plant nutrition and disease suppression (De la Fuente Cantó et al., 2020). Most of these microbes depend on plant exudates as a source of C for energy, and in exchange, they provide plants with essential nutrients like N, P, and other minerals that support plant health and growth (Singh et al., 2004).

The structure of the rhizosphere and endosphere microbial communities is influenced by a combination of abiotic factors, such as soil type, soil pH, and climate, and biotic factors, including plant species and root exudates (Berg and Smalla, 2009; Innes et al., 2004; Ma et al., 2019; Cavalieri et al., 2020). Among these, edaphic variables such as soil pH, soil organic matter, soil salinity, soil texture, and temperature play a dominant role in shaping the microbial community structures in the bulk soil and rhizosphere. As a result, distinct soil types may result in distinct soil microbial community structures (Schreiter et al., 2014; Bakker et al., 2015; Zhou et al., 2017). For example, it has been reported that acidic and alkaline soils generally have lower soil microbial diversity compared to neutral soils (Lopes et al., 2021; Zhang et al., 2015). An increase in organic material can enhance the diversity of both bacterial and fungal communities (Shu et al., 2022; Chen et al., 2024). Soil salinity stress can affect microbial functional genes (Dang et al., 2019; Zhang G. et al., 2021) and reduce the diversity of beneficial soil microbes (Rath et al., 2019; Zhang H. et al., 2021). Overall, soil pH is considered to have a significant influence on microbial structures in both the bulk soil and the rhizosphere.

It is also acknowledged that plant roots play a significant role in shaping the soil microbial community structure by releasing exudates and other labile chemical resources, which attract beneficial microbes and deter harmful microbes in the rhizosphere (Doornbos et al., 2012; Cavalieri et al., 2020; Chamkhi et al., 2022). Root exudates vary among plant species and are one of the main factors shaping the diversity of rhizosphere microorganisms associated with different plants. For example, benzoxazinoids exudated on maize promote *Pseudomonas putida* (Neal and Ton, 2013); isoflavones exudated on soybean roots promote *Bradyrhizobium japonicum* (Subramanian et al., 2006; Sugiyama, 2019); and glycogen exudated by carrots promote *Arbuscular mycorrhiza* (Salmeron-Santiago et al., 2021).

The foliar spray of glyphosate on GM crops may create selection pressure in rhizosphere and endosphere microbiota that could affect the availability of beneficial soil microbes (Reddy et al., 2004; Duke et al., 2012; Figure 1). This is because after the foliar application, a small amount of glyphosate is degraded within the GM crop by bounding to EPSPS, and the rest is translocated to metabolic sinks including the roots, and eventually released to the rhizosphere as root exudates (Kremer et al., 2005; Lane et al., 2012). After glyphosate is released from the roots, glyphosate residues encounter a diverse community of soil microbial species living in the rhizosphere and endosphere. Species such as *Bradyrhizobium japonicum* (Zablotowicz and Reddy, 2004; Fan et al., 2017; Amajioyi et al., 2023), *Pseudomonas* (Kremer and Means, 2009), Mn-reducing bacteria (Aristilde et al., 2017), and arbuscular mycorrhizal fungi (AMF) (Druille et al., 2013, 2016; Helander et al., 2018) experience metabolic disruptions after glyphosate exposure, because some are known to possess a sensitive EPSPS. These are beneficial microbial species that are ubiquitous in the rhizosphere and endosphere of most GM crops (e.g., in GM maize and soybean).

### 4.1 Glyphosate impacts on fungal communities

Among beneficial microorganisms in the rhizosphere and endosphere microbiota, fungal communities play a crucial role in promoting plant growth by enhancing nutrient cycling, particularly through the decomposition of organic matter and the release of essential nutrients like N and P. Fungi are the most abundant and diverse group of eukaryotes and their beneficial functions have been recognized for many years (Helgason and Fitter, 2009; Suman et al., 2022). Many fungal species are also valued for their biotechnological potential, synthesizing bioactive compounds that support plant development and acting as biocontrol agents against pathogens (Gange et al., 2019; Chitnis et al., 2020; Tao et al., 2023). Novel ecological functions, including epigenetic modifications and the suppression of virulence genes, further expand our understanding of strategies employed by plant-growth-promoting fungi. Their diverse roles as biofertilizers, bio-controllers, and biomodulators contribute significantly to sustainable agriculture and environmental resilience (Chitnis et al., 2020; Pozo et al., 2021). Within this diverse fungal community, AMF hold a special position due to their widespread association with plant roots and their critical role in improving plant nutrition and health, particularly under conditions of climate change and in soils with low fertility. However, despite the important plant growth-promoting functions of fungi, their contribution is often overlooked compared to that of bacteria (Zenteno-Alegría et al., 2024).

Under glyphosate stress, plant-growth-promoting fungi, including AMF, may be negatively impacted, disrupting the vital nutrient exchanges between plants and microbes colonizing the rhizosphere and endosphere. Glyphosate exposure can also alter fungal community composition, potentially decreasing the abundance of beneficial fungi while favoring pathogenic species that can supress plant health (Bünemann et al., 2006; Gómez Ortiz et al., 2017; Helander et al., 2018; Vázquez et al., 2021). Previously, Kremer et al. (2005) successfully demonstrated that after foliar spray of glyphosate on GM soybean, the roots showed high concentrations of carbohydrates and amino acids, while glyphosate significantly caused the growth of pathogenic fungi such as *Fusarium*. Means and Kremer (2007) also reported high colonization of *Fusarium* spp. after the foliar spray of glyphosate, where these fungal species can utilize the herbicide as a nutrient source (Krzyśko-Łupicka and Orlik, 1997). Interestingly, Meriles et al. (2006) demonstrated that glyphosate can promote the growth and sporulation of *Fusarium* spp. in soils amended with maize (*Zea mays* L.) or peanut (*Arachis hypogaea* L.) residues, even when glyphosate was not applied directly to the soil. These findings suggested that glyphosate may exert toxic effects through multiple pathways, including foliar application, direct soil exposure, and release from plant residues, which can promote pathogenic fungi such as *Fusarium* spp.

However, all these studies detected glyphosate effects focusing on specific genera or species such as AMF, *Bradyrhizobium japonicum*, *Fusarium*, *Pseudomonas*, and *Pythium* rather than with broader measurements of soil microbial diversity and functions (Kremer and Means, 2009; Zobiole et al., 2011; Aristilde et al., 2017; Helander et al., 2018; Vázquez et al., 2021). In addition, some studies that used basic methods such as phospholipid fatty acid (PLFA) profiles and molecular fingerprinting analysis of the bacterial and fungal genes such as denaturing gradient gel electrophoresis (DGGE) and terminal-restriction fragment length polymorphism (T-RFLP), reported that glyphosate had minor or no effects on soil microbial community structures (Hart et al., 2009; Allegrini et al., 2015; Cherni et al., 2015; Bruckner et al., 2019; Girgan et al., 2020). This can occur due to functional redundancy, niche partitioning, and horizontal gene transfer, where overall soil function remains stable despite microbial succession following glyphosate applications. Functional redundancy in microbial communities suggests that different species can perform similar ecological roles, such as N fixation or P solubilization, thereby buffering the ecosystem against disruptions caused by glyphosate exposure (Louca et al., 2018). Niche partitioning allows microbial species to occupy distinct ecological niches, with some developing specific resistance mechanisms to survive glyphosate stress (Zhang H. et al., 2021). Additionally, horizontal gene transfer can accelerate the spread of glyphosate resistance genes among microbial populations, leading to divergent community responses to herbicide pressure (Zhang G. et al., 2021). These factors highlight the need for future studies to employ advanced techniques that provide a more integrative and fine-scale analysis of soil microbial community structures, aiming to better understand the complex network of interactions between soil microorganisms and herbicides.

## 5 Metagenomic and bioinformatics approach

Next-generation sequencing (NGS), which involves the simultaneous sequencing of millions of DNA fragments, has revolutionized the microbial characterization along with development of bioinformatics tools by providing complete insights into genome structure, genetic variation, epigenetic modifications, and microbial diversity (Quince et al., 2017). Shotgun and targeted metagenomics are two NGS-based methods for functional and microbial characterization. In this review, our discussion will center on targeted metagenomics, which involves amplifying specific regions of the genome, and the utilization of bioinformatics tools for sequencing analysis to assign taxonomy (e.g., for archaea, bacteria, and fungi). In particular, the different platforms for targeted metagenomics, the databases (including their limitations) used in bioinformatics, and the contribution of these techniques in glyphosate-plant–soil-microbial research will be discussed.

### 5.1 Next-generation sequencing platforms: Illumina, Oxford Nanopore, and PacBio

To date, the commonly used sophisticated NGS platforms include Illumina, Oxford Nanopore, and PacBio (Lema et al., 2023). Illumina, which is known as second-generation sequencing, produces short-read sequences of 300 to 400 base pairs in length (Hu et al., 2021). Accordingly, Illumina platforms can identify short regions of the bacterial 16S rRNA gene and fungal ITS regions, which makes it difficult for species level identification. The archaeal, bacterial, and fungal composition of the complex microbial communities can be best determined at the genus level using Illumina platforms. However, the accurate identification of microbial communities at the species level is important in agriculture. One of the main reasons is that soil microbial communities identified at the genus level can contain both species of beneficial microbes and potential pathogens for each genus. For example, *Rhizobium* is mostly known to comprise species of beneficial N-fixing bacteria, but also contains species that are potential pathogens known to cause plant tumors (e.g., *Rhizobium rhizogenes*, *Rhizobium tumorogenes*, and Rhizobium nepotum) (Flores-Félix et al., 2020). Therefore, it may be challenging to detect potential pathogenic microbes in agricultural soil at the genus level rather than at the species level. However, with Oxford Nanopore and PacBio platforms, also referred to as third-generation technologies, soil microbial community composition can be identified up to the species level. This is because Oxford Nanopore and PacBio generates long-sequence reads of up to 10,000 base pairs, and this, for example, allows for the full-length sequencing of the bacterial 16S rRNA gene and fungal ITS regions (Heikema et al., 2020). However, due to the high costs associated with PacBio platforms (Rhoads and Au, 2015; Ardui et al., 2018), Oxford Nanopore platforms have emerged as a more cost-effective and widely adopted alternative (Cuber et al., 2023).

Previously, Oxford Nanopore technologies were not as widely adopted as Illumina technologies due to the high error rate in Oxford Nanopore sequencing, ranging from about 5 to 39% (Heikema et al., 2020), which impacts sequencing accuracy. The high error rate associated with the earliest versions of Oxford Nanopore technologies, particularly those using R9 and R10.3 flow cells, faced challenges related to signal resolution issues, single-strand signal noise, and difficulty in accurately reading homopolymeric regions (Delahaye and Nicolas, 2021; Sereika et al., 2022). Additionally, the absence of duplex (dual-strand) sequencing reduced basecalling precision, making it harder to differentiate true base signals from background noise. Current studies that use Oxford Nanopore Technologies still report an accuracy score of approximately 85 to 94% for sequence reads using MiniON sequencers and R9.4 (R9.4.1) flow cells (Winand et al., 2019; Mann et al., 2021; Miura et al., 2022), thus limiting the adoption of Oxford Nanopore Technologies. However, the introduction of the newest Oxford Nanopore, the R10.4.1 flow cell, can significantly reduce the error rate and reach an accuracy score of approximately 99% for the sequence reads (Sereika et al., 2022; Zhang et al., 2023). This is promising for glyphosate-microbial research in which Oxford Nanopore R10.4.1 flow cell can give an accurate resolution of soil microbial community species-level and be able to detect the exact species that are associated with glyphosate treatments.

### 5.2 Databases for metagenomics analysis

The taxonomic assignment is a crucial step in metagenomics analysis. Reference databases are required to transform raw sequence data obtained from NGS platforms into readable microbial names. Among the available reference databases, the SILVA (Yilmaz et al., 2014), and National Centre for Biotechnology Information (NCBI) (Federhen, 2012) databases are mostly used for taxonomic assignment. The taxonomic assignment through the SILVA database is based on phylogenies of short regions of the bacterial and archaeal 16S ribosomal RNA (16S rRNA) gene and fungal internal transcribed spacer (ITS) regions, which makes it suitable for sequence data from Illumina technologies (Table 1). The NCBI database, in contrast, is based on phylogenies of all organisms and can classify long-read sequence data. Therefore, it is predominantly utilized in the taxonomy assignment of sequence data from Oxford Nanopore and PacBio (Federhen, 2012). Some of the least utilized databases in targeted metagenomics include Greengenes and the Ribosomal Database Project (RDP), which provide 16S rRNA gene sequences for classifying short reads, and the UNITE database, which contains ITS region sequences and is also used to classify short reads (Table 1).

**Table 1:** Comparison of the most used reference databases in targeted metagenomics.

Databases	Commonly used platforms compatible	Recommended use	Targeted organisms	Commonly used classifiers and alignment tools	References
RDP	Illumina and Pyrosequencing	Classify short read sequences of 16S rRNA	Bacteria and archaea	Naïve Bayesian classifier (RDP classifier)	Cole et al. (2009) and Lan et al. (2012)
Greengenes	Illumina and Pyrosequencing	Legacy analyses of the 16S rRNA in older pipelines	Bacteria and archaea	SSU-ALIGN, QIIME, Mothur, and other tools	Weissbrodt et al. (2012) and Nygaard et al. (2020)
SILVA	Illumina	Classify short read sequences of 16S rRNA and fungal ITS	Bacteria, archaea, and eukaryotes	BLAST, DADA2, QIIME2, Usearch, and other tools	Nygaard et al. (2020) and Yilmaz et al. (2014)
UNITE	Illumina, Oxford Nanopore, and PacBio	Classify short read sequences of fungal ITS	Fungi	BLAST, QIIME2 DADA2, Usearch, and other tools.	Nilsson et al. (2019)
NCBI	Illumina, Oxford Nanopore and PacBio	Comprehensive genomic analyses and can classify both 16S rRNA and fungal ITS	All organisms	BLAST, QIIME2, Mininmap2, and other tools.	Schoch et al. (2020) and Cuber et al. (2023)

**Figure 3:**
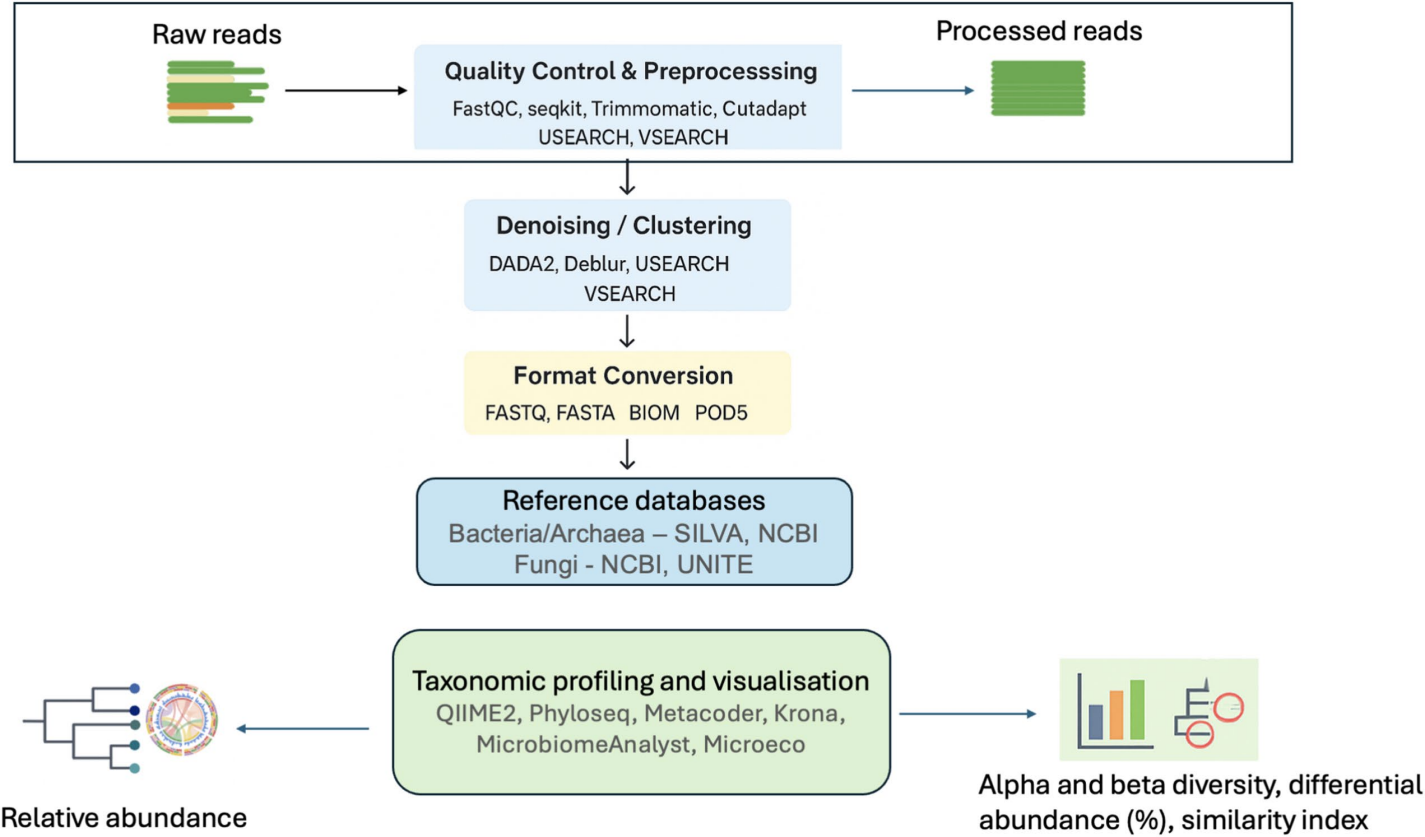
Taxonomy workflow for downstream analysis. Created with BioRender.com.

Despite the advancements and benefits of NGS technologies, errors and biases can still occur from computational analysis such as taxonomic assignment using databases and software implementation (Sierra et al., 2020; Zhang et al., 2023). After receiving the raw sequences from the NGS platforms, several bioinformatics tools are used for trimming the sequences and remove all non- biological nucleotides including DNA primers and sequence adapters. Then taxonomic classifiers and alignment tools (e.g., BLAST, QIIME2 DADA2, Mininmap2 etc., Table 1) are used to classify the sequence reads into readable microbial names using reference databases and determine the microbial diversity and taxa profiles (Figure 3).

It appears that a bias in the choice of the reference database compatible for the sequence reads obtained from Illumina, Oxford Nanopore, or PacBio may deeply alter microbial diversity, composition, and taxa profiles. For example, Sierra et al. (2020) used five different databases (Greengenes, the Human Oral Microbiome Database (HOMD), NCBI 16S, SILVA, and RDP) and two taxonomic Classifiers (QIIME and DADA2) to study the influence of reference databases in microbial diversity and taxonomic classification. The study showed that different reference databases in 16S rRNA amplicon data analysis leads to different microbial compositions, especially at the genus level. Ceccarani and Severgnini (2023) also found several inconsistent taxonomic classifications between Greengenes, SILVA, RDP, and NCBI databases. Balvočiūtė and Huson (2017) compared SILVA, RDP, and Greengenes with NCBI and OTT (Open Tree Taxonomy) by mapping their taxonomies and identified minor discrepancies. They further suggested that these differences could arise from variations in database sizes and the structural organization of the taxonomies. Furthermore, Zhang et al. (2023) demonstrated that the NCBI database, which contain sequences of all organisms produces higher correctly assigned rates for obtained reads than the SILVA database when using the newest Oxford Nanopore technology, R10.4.1 flow cell. As a result, this potentially indicates that the NCBI database is more accurate for taxonomic profiling when applied to NGS technologies producing long reads, especially in the analysis of environmental samples. These findings in the existing literature highlight the need to identify a standardized combination of NGS platforms, reference databases, and software tools to improve the resolution of microbial identification across various environments. The inconsistencies between reference databases make it challenging to directly compare results across studies, as they may depend on databases with varying levels of accuracy or annotation standards. As a result, these discrepancies in reference databases and software implementations could partially explain the contrasting results reported in glyphosate-microbial research, although differences in soil types, glyphosate rates, and GM crop varieties may also contribute to these inconsistencies.

In glyphosate-microbial research, there are limited studies utilizing NGS, especially with Illumina, Oxford Nanopore, and PacBio technologies (Vischetti et al., 2020; Zabaloy et al., 2022). The main reason could be the challenges in sequence data curation, which also complicate the comparison of results between studies for validating sequence annotations. For example, in this review, which appears to be the first of its kind, the results from glyphosate-plant–soil-microbial studies were compared by considering NGS platforms, databases, soil type, GM crop type, and glyphosate doses as the main factors influencing alpha diversity (*α*-diversity) measurements (chao1, abundance-based coverage (ACE), amplicon sequence variance (ASV), observed species richness, Shannon’s index, and phylogenic diversity) as well as microbial composition.

### 5.3 Next-generation sequencing in glyphosate-soil-microbial research

The bulk soil serves as the primary source of rhizosphere and endosphere microorganisms, as plant roots recruit microorganisms from the surrounding bulk soil (Massalha et al., 2017). Therefore, any changes in the bulk soil, such as those caused by the application of glyphosate in agricultural domains, will significantly alter the rhizosphere and endosphere microbiota in GM crops. As shown in Table 2, there are contrasting results about the impact of glyphosate on the richness and microbial composition in the bulk soil. Primarily, this could result from the fact that these studies used different soil types. In soils with high clay content, no significant glyphosate effects were detected on both soil microbial richness and soil microbial composition (Dennis et al., 2018; Guijarro et al., 2018; Table 2). Glyphosate sorption is very high in soils with high clay content, resulting in minimal glyphosate residues interfering with soil microbial communities (Duke et al., 2012). However, the use of different NGS platforms and reference databases between the studies may contribute to the inconsistent results between the studies. Considering NGS platforms and databases (Table 2), all the studies that used Illumina sequencing with the SILVA database, RDP database, and UNITE database for fungi, were able to detect significant effects regardless of the soil type used. For example, De Mesquita et al. (2023) detected significant glyphosate effects on bacterial and archaeal phylogenetic diversity and composition in bulk soil when using Illumina sequencing with the SILVA database, even though the soil contained high clay contents. This potentially suggest that the combination of NGS technology and the reference database has a greater effect on detecting microbial successions and changes in microbial richness and composition caused by glyphosate. Choosing a refence database compactable with the NGS platform can mitigate the challenges of receiving contrasting results between studies about glyphosate impacts on microbial communities.

**Table 2:** A systematic literature review of glyphosate impacts on microbial communities in bulk soil while utilizing next-generation sequencing (NGS).

NGS platform	Database	α-diversity after glyphosate application	Relative abundance of microbial communities after glyphosate application	Soil type	Glyphosate rate	References
Illumina	UNITE	For fungi, Shannon diversity decreased.	At the family level, *Ophiocordycipitaceae*, *Hypocreaceae*, *Nectriaceae,* and unidentified microbes increased.	Unspecified	1.7 kg ai ha^−1^	Schlatter et al. (2018)
Illumina	Greengenes	No significant effects.	No significant changes.	Clayey loam	9 Lha^−1^	Dennis et al. (2018)
Illumina	SILVA	The phylogenetic diversity decreased for bacterial and archaeal communities.	At the genus level, *Acidobacteria, Tychonema*, *Udaeobacter*, and *Nitrososphaera* increased.	Clayey loam	6 kg ai ha^−1^ and 0.7 kg ai ha^−1^	De Mesquita et al. (2023)
Illumina	Ribosomal Database Project (RDP)	The Chao1 and Shannon index for bacteria increased. Glyphosate had no significant effects on fungal diversity.	For bacteria, at the genus level, the relative abundance of *Sphingomonas* and *Phenylobacterium* increased. For fungi, at the genus level, *Talaromyces* and *Curvularia* were inhibited by glyphosate.	Sandy loam	0.6 mg ai kg^−1^ of soil	He et al. (2023)
Illumina	Unspecified.	No significant effects.	No significant changes.	Silty loam	10 Lha^−1^	Caggìa et al. (2023)
Pyrosequencing	SILVA	No significant effects.	No significant changes.	Loam	3 mg ai kg^−1^ of soil	Guijarro et al. (2018)
Pyrosequencing	SILVA	No significant effects.	At the family level*, Flammeovirgaceae* and *Saprospiraceae* (Sphingobacteriales-order) increased.	Clayey loam	3 mg ai kg^−1^ of soil	Guijarro et al. (2018)

At the genus level, glyphosate treatments in bulk soil increased some potential glyphosate degraders such as *Sphingomonas* and *Phenylobacterium* (He et al., 2023) and *Nitrososphaera* (De Mesquita et al., 2023), a N-fixing bacteria, while no pathogenic bacteria were detected in any of the studies. For fungi at the genus level, glyphosate decreased the abundance of *Talaromyces* (beneficial fungi) and *Curvularia* (mostly beneficial but can be pathogenic in plants) (He et al., 2023). Some studies have identified soil microbes up to the family level (Guijarro et al., 2018; Schlatter et al., 2018; Table 2), which complicates the comparison of taxonomic microbial composition between studies due to identifications made at different taxonomic ranks. Therefore, future studies should consider identifying microbial composition at a common taxonomic rank, especially at the species level, as this would facilitate the detection of specific pathogens encouraged by glyphosate.

### 5.4 Next-generation sequencing in glyphosate-rhizosphere-microbial research

Although there are new advanced techniques available to study microbial community structures, few studies have attempted to investigate the impacts of glyphosate on microbial communities in the rhizosphere of GM crops using NGS (Table 3). There are also contradictory results about glyphosate impacts on the richness and microbial composition in the rhizosphere of GM crops. These contradictory results also occurred due to the usage of different NGS platforms, databases, soil types, and GM crops between the studies as shown in Table 3. The studies that used Illumina sequencing together with the SILVA database detected significant glyphosate effects on microbial richness in the rhizosphere of GM crops (Fazal et al., 2023a; Schmidt et al., 2023; Table 3). A similar trend was observed in studies shown in Table 2 that used Illumina sequencing with the SILVA database, which could suggest the compatibility of Illumina and the SILVA database may be more sensitive in detecting glyphosate impacts on the soil microbial communities. However, Fazal et al. (2023b) also detected significant glyphosate effects on microbial richness in the rhizosphere of GM soybean when using PacBio technology with the SILVA database. This suggests that other NGS platforms, especially that produces long reads, may also be more sensitive in detecting glyphosate impacts on soil microbes. As a result, PacBio and Oxford Nanopore technologies are one of the sophisticated NGS platforms that should be applied in glyphosate-microbial research, as it may enhance our understanding of how NGS platforms and databases influence the detection of soil microbial communities affected by glyphosate treatments. Studies that successfully detected glyphosate effects demonstrated an increase in microbial richness in the rhizosphere of GM crops following glyphosate treatments (Fazal et al., 2023a, 2023b; Schmidt et al., 2023; Table 3). This resulted since most soil microbes utilize glyphosate as a food source (Zhan et al., 2018). At the genus level, the relative abundance of potential glyphosate degraders such as *Bacillus*, *Stenetrophomo-nas*, and *Pseudomonas* increased in the rhizosphere of GM crops following glyphosate treatments (Fazal et al., 2023b; Schmidt et al., 2023; Feng et al., 2024). Similarly, the relative abundance of N_2_ fixers such as *Bradyrhizobium*, *Rhizobium*, and *Devosia* also increased (Table 3). The identification of *Rhizobium* at the genus level, which may contain some potential pathogens at the species level such as *Rhizobium rhizogenes*, *Rhizobium tumorogenes*, and *Rhizobium nepotum*, highlights the importance of focusing more on finer taxonomic levels to understand whether glyphosate within this group encourages N_2_ fixers or potential pathogens. Where fungal communities are concerned, Fazal et al. (2023a) reported insignificant glyphosate effects on both richness and composition. This may suggest that bacterial communities are more sensitive than fungal communities in the rhizosphere of GM crops. However, to date, only limited studies have successfully identified fungal communities in bulk soil and rhizosphere of GM crops following glyphosate treatments following targeted metagenomics approach. This is due to the lack of a standardized and universally accepted method for identifying fungal communities in environmental samples (Lücking et al., 2020). Also, fungi have a diverse evolutionary history that vary depending on their lineage, which complicates their classification and identification.

**Table 3:** A systematic literature review of glyphosate impacts on microbial communities in the rhizosphere of GM crops while utilizing next-generation sequencing (NGS).

NGS platforms	Database	α-diversity after glyphosate application	Relative abundance of microbial communities after glyphosate application	GM crop	Soil type	Glyphosate rates	References
Illumina	SILVA	Bacterial species richness (ASVs) increased.	Genera *Bradyrhizobium*, *Bacillus*, *Rhizobium*, and *Cellvibrio* increased.	Soybean	Silty loam	0.87 kg.ha^−1^	Schmidt et al. (2023)
Illumina	Sequence Read Archive (SRA)	No significant effects.	No significant changes.	Soybean	Sandy loam	0.9 kg ai.ha^−1^	Yang et al. (2021)
Illumina	Greengenes	No significant effects.	Families *Xanthomonadaceae* increased and *Acidobacteria* decreased.	Maize	Silty loam	0.3 L.ha^−1^ (x2)	Newman et al. (2016)
Illumina	Greengenes	No significant effects.	Families *Xanthomonadaceae* increased and *Acidobacteria* decreased	Soybean	Silty loam	0.3 L.ha^−1^ (x2)	Newman et al. (2016)
Illumina	Ribosomal Database Project (RDP)	No significant effects.	The genera *Opitutus*, *Comamonas*, and *Dyella* decreased.	Soybean	Clay	3.9 L.ha^−1^	Lu et al. (2018)
Illumina	SILVA	Bacterial species richness (ACE and Shannon) increased. Insignificant effects on fungal species richness.	The bacterial genera *Bradyrhizobium*, and *Microbacterium* increased. No significant changes for fungal genera.	soybean	Sandy loam	0.9 kg ai ha^−1^	Fazal et al. (2023a)
Illumina	Not specified	Species richness decreased.	*Bradyrhizobium* and *Devosia* decreased, and *Pseudomonas* increased.	Wheat	Not specified	85 mg ai kg^−1^	Feng et al. (2021)
Pyrosequencing	SILVA	No significant effects.	No significant changes.	Cotton	Clay and Clayey loam	3 L.ha^−1^	Barriuso and Mellado (2012)
PacBio	SILVA	Bacterial species richness (observed, chao1, and Shannon) increased.	Genera *Bacillus* and *Stenetrophomonas* increased.	Soybean	Sandy loam	0.9 kg ai.ha^−1^	Fazal et al. (2023b)
Illumina	SILVA	No significant effects.	Beneficial bacteria with N fixation (*nifH*), and P solubilization (polyphosphate kinase, *ppk,* and alkaline phosphatase D *phoD*) genes increased.	Maize	Loam	1 kg per ha^−1^	Feng et al. (2024)

### 5.5 Next-generation sequencing in glyphosate-endosphere-microbial research

Plants selectively host specific microorganisms in their roots based on their beneficial functions, resulting in fewer microorganisms (endophytes) being present in plant roots. Glyphosate has been reported to negatively affect the symbiotic performance of microbial species in GM crops, as observed through culture-dependent approaches. For instance, culturable endophytes such as AMF and *Bradyrhizobium japonicum* have been primarily studied under glyphosate treatments and reported to exhibit decreased root colonization and functioning in GM crops (Kremer and Means, 2009; Zobiole et al., 2011; Aristilde et al., 2017; Helander et al., 2018; Vázquez et al., 2021).

To date, no studies have investigated microbial communities and richness in the endosphere of GM crops treated with glyphosate using a targeted metagenomics approach. This highlights the need for future research to explore both rhizosphere and endosphere microbiota to better understand the impacts of glyphosate on GM crops. Such studies could provide valuable insights into glyphosate-plant–soil-microbial interactions, offering a promising approach to mitigating glyphosate toxicity in agricultural domains. However, current limitations in targeted metagenomics, such as variations in reference databases and the limited use of long-read sequencing technologies like Oxford Nanopore and PacBio, hinder efforts to achieve high resolution of species-level identification. These challenges complicate conclusions about the toxicity of glyphosate on microbial communities in GM cropping systems. To address these issues, it is essential to develop standardized molecular and bioinformatics approaches that integrate long-read sequencing technologies and compatible reference databases (e.g., NCBI). Applying these methodologies to study microbial communities in bulk soil, rhizosphere, and endosphere microbiota could significantly enhance our understanding of glyphosate’s effects, ultimately offering a robust framework for mitigating its toxicity in agriculture.

In addition, the application of single-cell sequencing technologies offers a promising approach for studying microbial communities in the root endosphere at a strain resolution, even within the complex soil microbiome (Kifushi et al., 2024; Lengrand et al., 2024). By isolating individual microbial cells from plant roots and sequencing their genomes, this technique enables the identification and characterization of unculturable microorganisms that may play crucial roles in the plant-microbe interactions influenced by glyphosate. Moreover, combining single-cell approaches with traditional metagenomics can provide a more comprehensive understanding of microbial diversity and functional potential within the root endosphere (Lahlali et al., 2025), further advancing our ability to mitigate glyphosate toxicity in GM crops.

## 6 Conclusions and future perspectives

Glyphosate remains one of the most widely used herbicides globally due to its effectiveness in controlling weeds. However, its impact on soil microbial communities, particularly fungi, has become a growing concern. Fungi play a vital role in maintaining soil health by supporting processes such as nutrient cycling and disease regulation. Glyphosate application can alter fungal populations, affecting both their abundance and diversity. The extent of these changes depends on several factors, including glyphosate concentration, soil properties, and environmental conditions.

Higher doses of glyphosate typically reduce microbial biomass, whereas lower doses may temporarily stimulate microbial growth. The persistence of glyphosate residues in the soil further complicates this issue. These residues can interfere with microbial processes essential for nutrient cycling, particularly when glyphosate competes with phosphate for binding sites on soil particles, disrupting phosphorus availability. Recent advancements in metagenomics and bioinformatics have significantly improved our understanding of how glyphosate-based herbicides influence microbial communities, especially the balance between harmful and beneficial microorganisms in GM crops. Despite these advancements, inconsistencies across studies persist, mainly due to differences in experimental design, such as variations in soil type, glyphosate application rates, and the specific GM crops used. Additionally, differences in sequencing platforms and reference databases have made it difficult to draw definitive conclusions about glyphosate’s true impact on soil microbes. It remains unclear whether glyphosate promotes the growth of pathogenic species or supports beneficial microbes involved in nitrogen fixation and organic matter degradation.

To address these inconsistencies, future research should focus on implementing standardized bioinformatics approaches and integrating more accurate long-read sequencing technologies, such as PacBio and Oxford Nanopore, which provide more precise microbial identification at the species level. Moreover, there is a need to investigate less studied components of the soil microbiome, such as endophytic bacteria and fungal communities within both the rhizosphere and endosphere. These microbial communities are critical for developing bioremediation strategies to mitigate the ecological consequences of glyphosate use.

To gain a deeper understanding of how glyphosate affects plant-associated microbial communities, future studies should prioritize microbial populations within the endosphere. Methods like single-cell sequencing can offer detailed insights into microbial diversity within plant roots at the species level. Additionally, root-endophyte isolation techniques can help identify microorganisms directly associated with plant internal tissues, providing a clearer picture of how glyphosate impacts beneficial plant-growth-promoting microbes.

Future studies should also focus on improving microbial resilience in agricultural systems and further investigate long-term glyphosate’s broader impacts on microbial diversity. Implementing herbicide rotation strategies, such as alternating glyphosate with different herbicides or adjusting crop practices, can help manage weed resistance and reduce the pressure on soil microbial communities. This approach not only ensures continued glyphosate effectiveness but also enhances microbial diversity in the soil, ultimately promoting better soil health. Additionally, the use of beneficial microbial groups to clean up glyphosate-contaminated soils can restore microbial balance and mitigate its harmful effects. These microbes work together to degrade glyphosate and its byproducts. Integrating herbicide rotation with microbial treatments may not only maintain weed control efficacy but also promote microbial diversity, support remediation efforts, and ensure long-term sustainability in agriculture.
